# Multiple roles of neuronal extracellular vesicles in neurological disorders

**DOI:** 10.3389/fncel.2022.979856

**Published:** 2022-09-20

**Authors:** Zhigang Jiao, Zhengyi He, Nanhai Liu, Yanwei Lai, Tianyu Zhong

**Affiliations:** ^1^Laboratory Medicine, First Affiliated Hospital of Gannan Medical University, Ganzhou, China; ^2^Key Laboratory of Prevention and Treatment of Cardiovascular and Cerebrovascular Diseases, Ministry of Education, Gannan Medical University, Ganzhou, China; ^3^Precision Medicine Center, First Affiliated Hospital of Gannan Medical University, Ganzhou, China; ^4^Gannan Branch of National Geriatric Center, First Affiliated Hospital of Gannan Medical University, Ganzhou, China; ^5^Department of Clinical Research Center, The First Affiliated Hospital of Gannan Medical University, Ganzhou, China; ^6^Department of Neurology, First Affiliated Hospital of Gannan Medical University, Ganzhou, China

**Keywords:** neuron, extracellular vesicles, neuropathy, biomarker, neurodegeneration

## Abstract

Neuropathy is a growing public health problem in the aging, adolescent, and sport-playing populations, and the number of individuals at risk of neuropathy is growing; its risks include aging, violence, and conflicts between players. The signal pathways underlying neuronal aging and damage remain incompletely understood and evidence-based treatment for patients with neuropathy is insufficiently delivered; these are two of the reasons that explain why neuropathy is still not completely curable and why the progression of the disease cannot be inhibited. Extracellular vesicles (EVs) shuttling is an important pathway in disease progression. Previous studies have focused on the EVs of cells that support and protect neurons, such as astrocytes and microglia. This review aims to address the role of neuronal EVs by delineating updated mechanisms of neuronal damage and summarizing recent findings on the function of neuronal EVs. Challenges and obstacles in isolating and analyzing neuronal EVs are discussed, with an emphasis on neuron as research object and modification of EVs on translational medicine.

## Introduction

Neurons are the cells that make up the brain and the nervous system. They are the fundamental units that send and receive signals, allowing us to move our muscles, feel the external world, think, form memories, and much more. A great number of studies have shown that neurons are more vulnerable to various stresses, including oxidative stress, hypoxia, ischemia, inflammation, or neurotoxin than other neural cells and are considered less capable of regeneration and self-repair (Gonzalez et al., 2016). The disease burden resulting from neuronal damage induced by pollution, violence, and other factors is a growing health problem. Examples include Alzheimer’s disease (AD), Parkinson’s disease (PD), multiple sclerosis, amyotrophic lateral sclerosis (ALS), cerebral ischemia, cerebral hemorrhage, and traumatic brain injury, among others. The etiology and mechanism of neuron impairment are complex and remain to be elucidated. Numerous studies have shown that the dysregulation of cellular function underlying the process of neurodegeneration results from both abnormal cellular communication and misfolded proteins that cannot be effectively degraded or cleaned up. The exact mechanisms leading to these defects remain to be investigated (Peng et al., 2020), but studies have shown that major contributors are the classical pathway, including diffusion, phagocytosis, and exocytosis (Asai et al., 2015), and the unconventional secretion pathway, such as pore-mediated translocation, ATP-binding cassette transporter-based secretion, membrane-bound organelle-based secretion, the Golgi bypass pathway, and extracellular vesicles (EVs) secretion.

EVs have recently caught more attention due to their physicochemical characteristics and biological function. EVs are one type of small lipid bilayer vesicles, including large vesicles (in the size range of ∼50 nm to 1 μm) and exosomes [in the size range of ∼40–160 nm (average ∼100 nm in diameter)] and have an important role in the therapy and development of neurodegenerative diseases. Although many research approaches on EVs have been established and applied, it is challenging to study alone the large vesicles or exosomes due to the resemblance of their biogenesis pathway and regulatory mechanisms. Therefore, in this manuscript we favor the term EVs.

EVs were first observed and termed “platelet dust” 50 years ago (Wolf, 1967). EVs contain many cell components, depending on the original cell, including nucleic acid, lipids, metabolites, cytosolic and cell-surface proteins, and can be found in all biological fluids and tissues, including the central nervous system (CNS). However, the physiological purpose of generating EVs remains largely unknown and needs to be investigated. One speculated role is that EVs remove excess and/or unnecessary constituents from cells to maintain cellular homeostasis, a process that would be similar to autophagy, which was discovered earlier and is also considered to eliminate the cellular garbage (Mizushima and Komatsu, 2011; Babuta et al., 2019). Meanwhile, studies in these years found that both processes play a vital role in cellular homeostasis and intercellular communication (Pan and Johnstone, 1983; Harding et al., 1984) and affect each other through common elements (Babuta et al., 2019; Zhang X. W. et al., 2020). Notably, studies indicated a negative correlation between the ratio of the release of EVs with the ratio of formation of autophagosome—the intermediate material of the autophagy process (Zhang X. W. et al., 2020), and the EVs biogenesis and release are closely related to the formation of the multivesicular body (MVB), which is also involved in autophagy (Baixauli et al., 2014). These studies supported the conclusion that EVs may play a role in the process of cellular homeostasis.

Recent studies reviewed here also indicate a functional, targeted, and active accumulation of specific cellular components in EVs, suggesting that they may play an important role in regulating intercellular communication and trigger phenotypic changes in the recipient cells (Pegtel and Gould, 2019). For instance, EVs from nicotine-stimulated macrophages promote the migration and proliferation of vascular smooth muscle cells (VSMC), whereas neuron-derived EVs promote the polarization of M1 microglia in culture (Zhu et al., 2019; Yin et al., 2020). In addition, several lines of evidence showed that EVs from CNS might directly alter the function of the nearby cell *via* the paracrine pathway or leave the brain and betray the state of CNS as potential biomarkers released into the biological fluids, including cerebrospinal fluid (CSF), plasma, serum, saliva, urine, semen, breast milk, amniotic fluid, and ascites fluid (Wang and Zhang, 2020; Yu et al., 2020). In the meantime, many studies revealed that EVs derived from neurons and/or glial cells might be involved in the initial development of neurodegeneration. However, the culprit of neurodegenerative diseases remains to be investigated (Singh and Muqit, 2020; Yin et al., 2020; You et al., 2020). This review focuses on the regulation mechanism of biogenesis, the release of the neuronal EVs, and its effects on the receptor cells in the pathogenesis, diagnosis, treatment, and prognosis of neurodegeneration. Glia-derived EVs and their effects in neurons have been the focus of several studies and will not be addressed in this review (Lemaire et al., 2019; Upadhya et al., 2020).

## Biogenesis and release of neuronal extracellular vesicles

**FIGURE 1:**
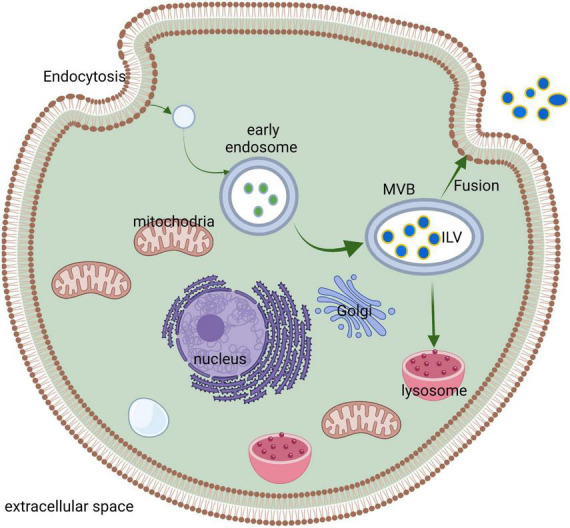
A common process for EVs synthesis and release. Cells internalized the cellular components/extracellular ligands and then formed the early endosome. Subsequently, the early endosome matured into MVB, and the inward budding of early endosomes formed ILVs. In the last step, MVB either digests and degrades after fusion with lysosomes or releases exosomes through fusion with cell membranes.

The biogenesis and release of neuronal EVs are partially similar to other cells (Figure 1). For example, the biogenesis of neuronal EVs also begins with the endosome system and is released into the extracellular space through fusion with the plasma membrane (Hessvik and Llorente, 2018). However, this mode is not the only pathway due to multiple lines of evidence showing that EVs also directly bud from the plasma membrane (Nabhan et al., 2012; Bianchi et al., 2014; Nager et al., 2017). The general process of EVs biogenesis and release mainly include three steps: the first step, the formation of early endosome mediated by endocytosis; the second step, the production of intracellular MVBs regulated by endosomal sorting complexes required for transport (ESCRT) machinery, including ESCRT-0, ESCRT-I, ESCRT-II, and ESCRT-III (Hessvik and Llorente, 2018) or ESCRT-I, ESCRT-II, and ESCRT-III (Vietri et al., 2020), with their accessory proteins and then the intraluminal vesicles (ILVs) formed by inward budding from the limiting membrane of MVBs; and the last step, in which ILVs are secreted through the MVBs containing ILVs fused with the plasma membrane and exocytosis.

In general, the ESCRT machinery is considered to play a vital role during the assembled MVBs and the budding of ILV (Colombo et al., 2013; Vietri et al., 2020). However, the pathway of independent-ESCRT is also uncovered, such as the interaction of RAB31 with the epidermal growth factor receptor (EGFR) (Wei et al., 2021) or the tetraspanin pathway (Kenific et al., 2021). Indeed, loss of VPS4 function, an essential factor for the ESCRT function (Vietri et al., 2020), did not affect the secretion of EVs marker proteins such as CD63 (Fang et al., 2007; Trajkovic et al., 2008; Colombo et al., 2013). These studies demonstrated that other molecules might regulate EVs biogenesis beyond the ESCRT machinery. In addition, evidence collected during the last 20 years also indicated that the dynamics of protein trafficking are also involved in the process of EVs biogenesis and release. However, the regulatory mechanisms of these processes remain to be elucidated due to its high precision and complexity (Pegtel and Gould, 2019). For instance, a vast number of studies found that the Ras-related protein GTPase Rab (Ostrowski et al., 2010), Sytenin-1, tumor susceptibility gene 101 (TSG101), apoptosis-linked gene 2-interacting protein X (ALIX), syndecan-1 (Baietti et al., 2012), ESCRT proteins (Colombo et al., 2013), tetraspanins (Escola et al., 1998), ceramides, sphingomyelinases, and soluble N-ethylmaleimide–sensitive factor attachment protein receptor (SNARE) complex proteins (Vilcaes et al., 2021) are involved in the origin and biogenesis process of EVs (D’Souza-Schorey and Schorey, 2018; Pegtel and Gould, 2019). However, their precise rate-limiting actions and functions in EVs biogenesis require further in-depth exploration, especially *in vivo*.

The Rab protein family has received more attention due to their roles in regulating vesicle trafficking between different compartments along the endo-lysosomal and secretory pathways (Pfeffer, 2017). For example, studies found that Rab27, Rab35, or Rab11 cooperation with their effectors Slp4, Slac2b and Munc13-4, and regulator TBC1D10B play important roles in MVB biogenesis and positioning. The loss of Rab27 or Rab35 function results in a ∼50–75% drop or little effect on EVs biogenesis upon differentiation of a cell line (Hsu et al., 2010; Ostrowski et al., 2010), overexpressing Rab11 within RAW macrophages elevated the amounts of EVs by about 50% through promoting homotypic fusion/maturation of MVBs (Savina et al., 2002). It is worth noting that there is controversy about how Rab proteins regulate EVs biogenesis. For instance, studies have shown that Rab27/Rab11 proteins promote MVB maturation and deliver MVB to the plasma membrane (Savina et al., 2002; Ostrowski et al., 2010). Others revealed that Rab27a is also required for assembling plasma membrane microdomains (Booth et al., 2006). Thus, it appears that Rab27 proteins control exosome biogenesis at both endosome and plasma membranes. In addition, Ostrowski et al. (2010) found that inhibition of Rab11a did not significantly modify EVs secretion in the HeLa B6H4 cell line. These results indicated that EVs biogenesis and secretion, which do not have to comply with the canonical regulations, were regulated by multiple pathways.

Although most of our knowledge is about the mechanism of EVs biogenesis and secretion in cancer (non-neuronal) cells, many of these mechanisms also function in neurons. However, it is known that the regulatory mechanism of EVs biogenesis and secretion depends on the cell type and state. Therefore, both processes of neuronal EVs have a different pathway compared to cancer cells due to the differential expression of genes and their extreme morphologies. For example, previous research found that endocannabinoid 2-arachadonoylglycerol (2-AG) containing EVs derived from midbrain dopaminergic (DA) neurons can be released in the Arf6-myosin light chain kinase (MLCK) pathway (Nakamura et al., 2019). However, the Arf6-MLCK pathway is specifically attributed to microvesicle (MV) release in tumor cells (Muralidharan-Chari et al., 2009). Moreover, neuronal EVs are released in response to stimulation or depolarization. Furthermore, a positive correlation was observed between the concentration of neuronal EVs and the degree of neuronal depolarization (Faure et al., 2006; Blanchette and Rodal, 2020). Meanwhile, previous studies suggest that neuronal somatodendritic compartments or axons may be the release site of neuronal EVs (Coulter et al., 2018; Polanco et al., 2018). However, the above-mentioned correlation or similar bioconstructors remain to be explored in cancer cells. The same genes may even play different roles in regulating the biogenesis or release of EVs depending in different types of cells. For instance, knockdown of the autophagy-related gene 5 (*ATG5*) in the MDA-MB-231 cells (a human breast cancer cell line) not only inhibited the autophagy but also induced a severely decreased EVs production (Guo et al., 2017). In contrast, knockout of *ATG5* in neuronal cells greatly increases the release of EVs and EV-associated prions from neuronal cells (Abdulrahman et al., 2018). This result showed that the regulatory mechanism of EVs biogenesis and release is not common.

Due to above reasons, studies about EVs derived from neurons need to have neurons as their research object rather than other cell lines such as HEK293 (Zheng et al., 2017, 2018). The exciting news is that some experiments using neurons as research objects have been implemented and acquired some achievements in healthy or disease models. For example, a study in hippocampus neurons found that brain fibroblast growth factors-2 (bFGF2) can greatly improve the fusion of MVB with the plasma membrane and the release of neuronal exosome by promoting the v-SNARE vesicle-associated membrane protein 3 (VAMP3) translocation to the exosome rather than VAMP2 (Kumar et al., 2020). Moreover, the bFGF2 system regulates neuronal calcium homeostasis and plasticity and promotes neuroprotection and repair in response to neural tissue damage (Guillemot and Zimmer, 2011). Blocked Drp1 in N27 or SH-SY5Y cells treated with PFF (Human α-synuclein preformed fibrils) can enhance the autophagy flux, inhibit the release of EVs, and prevent the transmission of aggregative or misfolded α-synuclein (α-syn) protein between neurons *via* EVs (Fan et al., 2019). KIBRA regulates the secretion of EVs derived from the mouse hippocampal and cortex neuron rather than the liver or muscle tissue *via* inhibiting the proteasomal degradation of Rab27a (Song et al., 2019c). Although the function of these proteins or genes may also be involved with other disorders and needs to be investigated, it has been confirmed that the disorder of these proteins or their gene expression is involved in the progress or development of neurodegeneration such as major depressive disorder, bipolar disorder (BPD), PD, or AD (Evans et al., 2004; Gaughran et al., 2006; Song et al., 2019b; Zhang W. et al., 2019). Therefore, based on the above results, we suggested that disorders in the neuronal EVs biogenesis or release contribute to the neurological disorder. More interestingly, Pastuzyn et al. (2018) found that neuronal Arc protein self-assembles into virus-like capsids that encapsulated *Arc* mRNA and mediated the transmission of Arc protein and *Arc* mRNA between neurons in EVs, where it can undergo activity-dependent translation to regulate the synaptic plasticity of recipient neurons. This finding provides new insight into EVs biogenesis or release mechanism. Moreover, the secretion of neuron-derived EVs was also modulated by the activities of sphingolipid metabolizing enzymes, including neutral sphingomyelinase 2 (nSMase2) and sphingomyelin synthase 2 (SMS2) (Yuyama et al., 2012); however, nSMase2 or SMS2 expression can be detected in other cells (Hitomi et al., 2020), the regulated biogenesis or secrete mechanism of EVs by nSMase2 or SMS2 in other cells are few.

## Neuronal extracellular vesicles affected the function of glial cells and other cells

EVs derived from neurons are continuously secreted into the extracellular space and are up taken by the glia cells and nearby neurons or other cells through neuronal junctions (Faure et al., 2006). The cargo of EVs, like non-coding RNAs (ncRNAs) or proteins and other molecules, influences the function of recipient cells by binding their target molecules. Although how and why neuronal EVs are delivered into other cells are being investigated, some studies have shown that the neuronal EVs containing ncRNA or proteins may modulate the neuronal survival or activity, neural circuits, and the function of other organs by regulating the specific gene expression of receptor cells. For example, Morel et al. (2013) found that exosome-containing *miRNA-124a* derived from a cortical neuron can be engulfed by astrocytes and indirectly regulate glutamate transporter-1(GLT-1). Furthermore, they confirmed that the translational regulation of GLT-1 protein in astrocytes is dependent on neuronal exosome-containing *miRNA-124a* (Morel et al., 2013). However, they did not identify the target genes of *miRNA-124a* that regulate the translation of the GLT-1 protein in astrocytes. Men et al. also confirmed that secreted neuronal exosome-containing miRNAs could be shuttled into astrocytes and influence the function of astrocytes (Men et al., 2019). Furthermore, overexpressed *miRNA-124-3p* in exosomes can strongly upregulate the expression of GLT-1 *via* the abolition of the inhibitory effects of *miR-132* or *miR-128* on GLT-1 in astrocytes (Men et al., 2019). Our previous studies indicated that downregulation of GLT-1 expression in astrocytes results in the drastic increase of glutamate concentration in the dopaminergic (DA) neuronal synaptic cleft and then induces DA degeneration *via* glutamate excitatory toxicity (Zhang Y. et al., 2020). There is also evidence indicating that *miRNA-124-3p* is involved in the perioperative neurocognitive disorders in patients after cardiac surgery (Xiao Q. X. et al., 2020). Furthermore, neuron axonal exosome-containing *miRNA-124-3p* can suppress the activation of M1 microglia and A1 astrocyte *via* inhibiting MYH9 by directly targeting its 3′-UTR and thereby modulating the PI3K/Akt/NF-κB pathway improving over the motor function of spinal cord injury (SCI) mice (Jiang D. et al., 2020). However, the exact role of *miRNA-124-3p* in neurodegenerative diseases remains to be investigated.

The effects of EVs on recipient cells depend on the state of neuron. EVs derived from the neurons with physiological status or drug treatment may protect other cells. For instance, healthy cortical neurons released the *miRNA-181-3p*-containing exosome, which suppressed the neuroinflammation by targeting the CXCL1 gene in astrocytes. However, the expression of neuronal and exosomal *miRNA-181-3p* is significantly elevated in the ischemic brain injury (IBI) (Song et al., 2019a). CD2019 improved the deficiency of locomotor and sensory *via* promoting the regeneration of neuronal axonal and decreasing the glial scar formation in SCI rats. The underlying mechanism of the protective effect may be the induction of the translocation of PTEN and promotion of the secretion of enriched PTEN-containing exosome *via* activating the retinoic acid receptor β (RARβ). These PTEN-containing exosomes are then delivered to astrocytes, inhibiting the proliferation and activation of the cells (Goncalves et al., 2015). In contrast, the gene expression in damaged neurons causes the dysregulation of the cargo that is packaged into EVs. Subsequently, these cargo molecules are delivered to other cells and then disrupt the function of recipient cells. For example, Yin and his colleagues found that the expression of *miRNA-21-5p* is significantly elevated in PC12 cells and exosomes from PC12 cells after scratch injury. These EV-containing *miRNA-21-5p* shuttle into the BV2 cells, inducing the polarization of cells, and then the polarized BV2 cells cause chronic neuro-inflammation *via* secreting pro-inflammation factors like IL-1β, IL-6, and TNF-α (Yin et al., 2020). Irreversible neuro-inflammation is considered to play an important role in the onset and development of neurodegenerative disease (Calsolaro and Edison, 2016; Han X. et al., 2019; Jiao et al., 2019; Passamonti et al., 2019). SH-SY5Y cells transfected with wild-type human *SNCA* gene secreted exosomes with a higher expression of *miRNA-19a-3p*. The *miRNA-19a-3p*-containing exosomes elevated the expression of *miRNA-19a-3p* in microglia *via* delivering *miRNA-19a-3p* into the microglia, and then the miRNA inhibited the autophagy of microglia by targeting the PTEN/AKT/mTOR signal pathway (Zhou T. et al., 2019). However, a healthy autophagy-lysosome pathway of microglia plays a crucial role in the process that cleans up toxic molecules such as misfolded α-syn (Nash et al., 2017; Choi et al., 2020), abnormal tau, amyloid-β (Aβ) protein (Cho et al., 2014; Heckmann et al., 2019; Houtman et al., 2019), and cellular debris (Pluvinage et al., 2019). On the contrary, the dysregulated autophagy of microglia impacted the degradation of phagocytized cargo and induced an accumulation of misfolded proteins or cellular debris in the microglia or extracellular space, which ultimately contributes to the pathophysiology of neurodegeneration (Qin et al., 2021).

**FIGURE 2:**
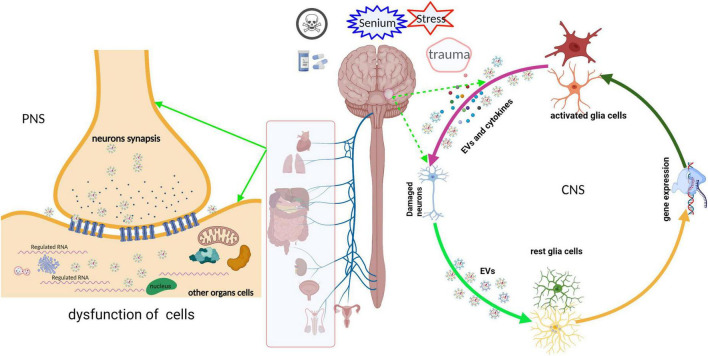
Schematic representation of the neuronal EVs that regulated the function of receptor cells. EVs derived from neurons undergoing various stress deliver their cargoes, such as ncRNA, mRNA, or misfold proteins, into other cells in CNS or PNS *via* paracrine or active transport. These cargoes are abnormal expressions and are packaged into EVs, which disturb the gene expression or signal pathway of the recipient cells, ultimately leading to cell dysfunction. Meanwhile, dysfunctional recipient cells secrete “detrimental molecules” and then damage the neurons or nearby cells, and vice versa.

Neuronal EVs in CNS may be absorbed by glial cells; however, neuronal EVs of the peripheral nervous system (PNS) regulated the function of other cells by shuttling to other organ cells and neurites (Figure 2). For instance, the impaired sensory neuron in dorsal root ganglia secreted exosomes with a high *miRNA-21* expression that macrophages may engulf by phagocytosis, inducing their activation and release of pro-inflammation cytokines (Simeoli et al., 2017). These cytokines recruit more macrophages, which infiltrate into the site of injury, amplifying and deteriorating the immunity response, ultimately causing neuro-inflammation and neurodegeneration (Harland et al., 2020; Zhang Y. et al., 2020). Xu et al. (2017) also found that neurons transfer *miR-132 via* secreting exosomes to endothelial cells (ECs) and then regulate the expression of vascular endothelial cadherin (VE-cadherin), an important adherens junction protein, by directly targeting the eukaryotic elongation factor 2 kinase (eef2k) to maintain brain vascular integrity. However, an adequate supply of blood and structural and functional integrity of blood vessels are key to normal brain functioning. In contrast, cerebral blood flow shortfalls and blood-brain barrier dysfunction can be earlier discovered in neurodegenerative disorders in humans and animal models (Iturria-Medina et al., 2016; Sweeney et al., 2018; Zhang C. et al., 2020). The abnormal structure and function of blood vessels result in the destruction of blood-brain barrier integrity, and peripheral immune cells like T cells infiltrated into CNS and evoked neuro-inflammation. For instance, evidence from postmortem or animal models of neurodegenerative disorder showed that T cells are abundantly present in the impaired region of the brain (Machado-Santos et al., 2018; Liu et al., 2019; Lodygin et al., 2019; Haque et al., 2020). Meanwhile, we reported that the expression of *miRNA-132* is significantly reduced in primary hippocampus neurons treated by soluble Aβ oligomers, a classical cell model of AD (Wei et al., 2020). Furthermore, oxidative stress after SCI injured neurons and altered the release of the neuronal exosome; secreted exosomes promoted the apoptosis of transplanted bone marrow-derived stem cells with oxidative stress (Luo et al., 2019).

In addition to ncRNA, proteins such as Tau and α-syn within EVs also regulate the function of recipient cells and even disturb the cellular homeostasis (Meng et al., 2020). For example, NSC-34 cell, a kind of motor neuron-like cell, mutated superoxide dismutase 1 (mSOD1, G93A) as donor cells released exosome-containing mSOD1, which are phagocytosed by N9-microglial cells. Internalized exosome-containing mSOD1 upregulated the expression of IL-1β, TNF-α, MHC-II, and iNOS gene *via* activating the NF-κB pathway in N9 cells and reducing the phagocytic ability of N9 cells (Pinto et al., 2017). Meanwhile, neuronal PTEN can be transferred to astrocytes by EVs and inhibit the information of glia scar (Goncalves et al., 2015). The formatted glial scar is considered an obstacle to the regeneration of neuronal axons (Anderson et al., 2016). Tau pathology or misfolded α-syn protein spreading is also achieved by direct transmission of exosomes between neurons and then induced Tau protein aggregation or misfolded endogenous α-syn in the receptor neurons (Wang et al., 2017; Sardar Sinha et al., 2018); ultimately, these abnormal proteins induced neurodegenerative diseases.

Based on the above results, we proposed that the expressed dysregulation encapsulated in neuronal EVs containing ncRNAs or pathological proteins induced by diverse factors, such as neurotoxic or mild traumatic brain injury, may result in neurodegeneration. It is noteworthy that almost all studies supposed that the level of cargo in EVs depends on the parental cells. However, Men et al. (2019) found that secreted neuronal exosomes contain a subset of microRNAs that is drastically different from the miRNA profile of neurons. This result is significantly distinct from previous reports (Morel et al., 2013; Xu et al., 2017; Ying et al., 2017; Zhu et al., 2019). Therefore, the sorting mechanism of exosomal cargo remains to be investigated.

## Neuronal extracellular vesicles modulated the function of other neurons

**FIGURE 3:**
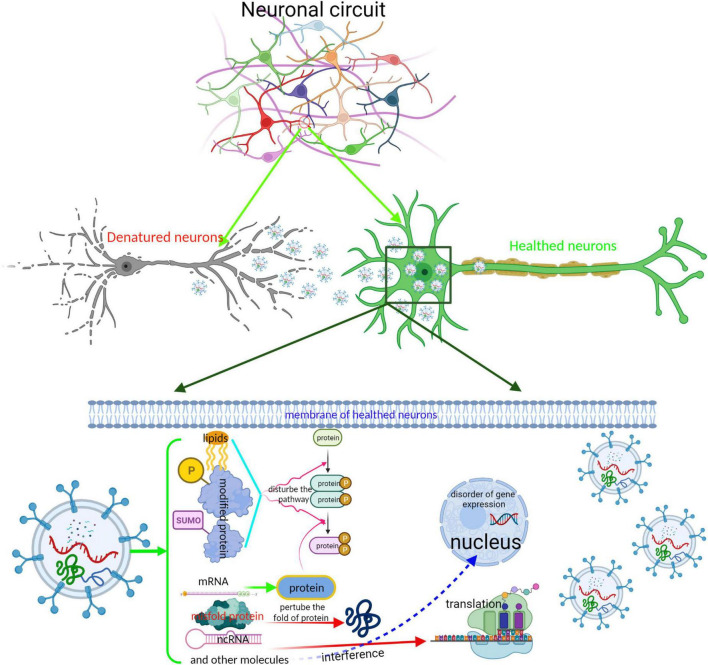
Schematic representation of the interplay between neurons by EVs. EVs is an important tool for maintaining the completion of neuronal circuit and the delivery of neurotransmitters. EVs derived from impaired neurons containing abnormal regulators or neurotransmitters are absorbed into healthy neurons, perturbing the neuronal function. The completion of neuronal circuit depends on the neuronal function. Especially, those EVs containing misfold proteins, such as Tau, α-syn, and TDP43, spread to other neurons; these misfold proteins acting as “pathological seeds” induce misfolding of endogenous protein, cause a dissemination from local brain region to others, and accelerate disease progression.

The postmortem studies identified that the stereotypical and temporal distribution of pathology is limited due to a lack of longitudinal data and therefore do not provide direct evidence of the sequential evolvement of different brain regions during disease progression. Nevertheless, numerous studies from cell and animal models have shown that pathological proteins such as α-syn and Tau, or the abnormal regulator such as miRNA, lncRNA, and rRNA undergo neuron-to-neuron transmission and partially contribute to the progress of neurodegenerative diseases (Emmanouilidou et al., 2010; Zhou W. et al., 2019; Ruan et al., 2021; Vilcaes et al., 2021). Although the modes of interplay with neurons are various, EV-based secretion and uptake, one of the unconventional secretion pathways, is the most extensively studied pathway for the transmission of excessive regulators of pathological proteins. EVs containing an excessive level of regulators or misfolded proteins are engulfed into the nearby or distal neurons. The former induced a dysfunction in neurons by disturbing the expression of a specific gene in the recipient neurons. The latter work as “pathological seeds” that damage the function of neurons *via* inducing their normal endogenous counterpart protein to misfold, leading to the amplification of the pathological protein conformation in the receptor cell (Wang et al., 2017; Figure 3). Previous research has revealed that EVs derived from dysfunctional neurons may contain the “misfolded proteins” such as α-syn oligomer in PD (Niu et al., 2020), Aβ and tau aggregates in AD (Guix et al., 2018; Sardar Sinha et al., 2018), TAR DNA-binding protein 43(TDP43) and mSOD1 pathology in ALS (Neumann et al., 2006; Iguchi et al., 2016; Silverman et al., 2019), and mutated huntingtin (HTT) (DiFiglia et al., 1997; Jeon et al., 2016). These “unusual EVs” are taken up by other neurons and disturb cellular homeostasis by perturbing the gene expression or protein folding. For example, Han et al. found that the intravenous or intrastriatal treatment of mice with exosomes derived from patients with PD containing α-syn, IL-1β, and TNF-α could trigger dopamine neuron degeneration *via* inducing α-syn, ubiquitin, and p62 aggregation in recipient cells (Han C. et al., 2019). Sinha et al. also found that tau-containing exosomes derived from SH-SY5Y cells when treated with Aβ shuttled to the normal SH-SY5Y cells and increased the LDH level of normal SH-SY5Y cells; dynasore, which is an inhibitor of endocytosis, can inhibit this cytotoxicity effect (Sardar Sinha et al., 2018). Furthermore, Park et al. also confirmed that the Fas-associated factor 1 (FAF1) of SY5Y cells was secreted and transmitted to neighboring cells *via* exocytosis as well as an exosomal pathway, where it induced cell death through apoptotic and necrotic pathways (Park et al., 2020).

The dysfunction of a neuron may cause the disorder of package and release of the exosome. For example, studies have shown that the release of cortex neuronal exosome is regulated upon the calcium homeostasis in the cytoplasm and glutamatergic synaptic activity (Faure et al., 2006; Lachenal et al., 2011); however, both contribute to neuronal impairment (Cheng et al., 2016; Malik and Willnow, 2019; Alvarez et al., 2020). In contrast, the cargo of normal neuronal EVs contribute to the homeostasis of neuronal function and circuit. For example, Sharma et al. found that exosomes derived from hiPSC-derived neurons increased neurogenesis by promoting cultured primary neuronal proliferation and differentiation and rescued the aberrant neural circuit development mediated by X-linked *Methyl-CpG-binding protein 2* (*MECP2*) gene mutation, which is involved with Rett syndrome (Sharma et al., 2019). Although their study did not reveal the effector within the exosome, these results confirmed the protected role of exosomal cargo in neurons. Subsequently, studies indicate that miRNA-containing exosomes derived from normal cells play a protective role in maintaining the function of other cells (de Rivero Vaccari et al., 2016; Mead and Tomarev, 2017). For instance, HT-22 secreted *miRNA-21-5p*-containing exosomes exert a protection effect by targeting the Rab11a to inhibit the autophagy flux of injured HT-22 (Li et al., 2019). Furthermore, *miRNA-126-3p*-enriched EVs from hypoxia-preconditioned VSC 4.1 neurons attenuate ischemia-reperfusion-induced pain hypersensitivity by regulating the PIK3R2-mediated pathway (Wang et al., 2021). Together, these papers uncovered the partial underlying mechanism of the gradual progress of neurodegeneration and confirmed the role of neuronal EVs in the disease process. Meanwhile, they also verified that the interplay between neurons is vital for maintaining neuronal homeostasis except for the glial cells which deliver nerve trophic factor into neurons (Upadhya et al., 2020) or maintain the synaptic plasticity of neurons using EVs (Wilton et al., 2019).

## The implication of neuronal extracellular vesicles in the diagnosis and therapy of neuropathy

**FIGURE 4:**
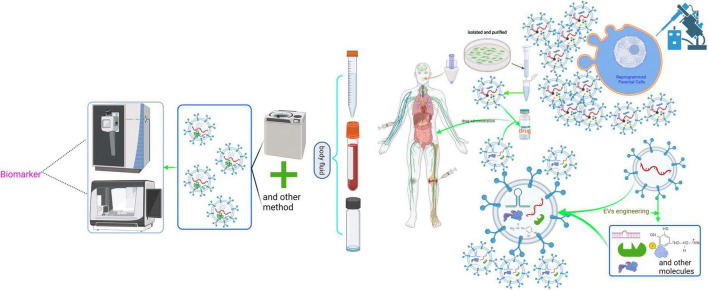
Schematic representation of the clinical application of neuronal EVs. The cargoes in EVs are derived from the parental cells, which partly reflect the physiological or pathological state of the donor cells. Moreover, EVs can effectively protect their contents from being degraded by various enzymes in body fluids, prolonging the half-life of various biomarkers. Therefore, isolating and purifying EVs from body fluids using various methods, including ultracentrifugation, and then analyzing and studying them are of great significance for predicting the state of cells. However, clinical application of EVs is still quite difficult because there is no effective method to purify EVs from a defined cell. Additionally, based on the physical and chemical characteristics of EVs, such as the ability to pass the blood-brain barrier, EVs can package some targeted drugs *via* genetic manipulation to deal with neuropathy.

EVs are released into various body fluids or across the various biological membranes into the cells due to their physicochemical characteristics, such as the small size and similar composition with the cellular membrane. Meanwhile, EV contents are cellular-specific due to the existence of different donor cells. Furthermore, EVs secretion is a constitutive phenomenon involved in both physiological and pathological processes. Hence, many studies focused on the EVs and their carried cargo as biomarkers, vaccines, and drug carriers, and have modified them rationally for therapeutic interventions (Figure 4). In particular, the development of EVs for cancer is exploding (Rayamajhi et al., 2019; Zhang W. et al., 2019; Guo et al., 2020; Nie et al., 2020; Xiao Y. et al., 2020; Liu et al., 2021; Yang et al., 2021; Zhao et al., 2021). It is worth mentioning that the development of exosome-containing GPC-1(GPC-1^+^ crExos) for pancreatic ductal adenocarcinoma (PDAC) is inspiring. For example, Melo et al. (2015) found that GPC-1^+^ crExos derived from PDAC and breast cancer patients’ serum is significantly elevated. They found that GPC-1^+^ crExos strongly correlates with the PDAC rather than breast cancer. The most impressive finding was that the GPC-1^+^ crExos distinguished patients with PDAC and pancreatic cancer precursor lesions (PCPL) from healthy donors and BPD patients; the sensitivity and specificity of GPC-1^+^ crExos prediction are 100% compared with CA19-9 (the clinical standard tumor biomarker for patients with PDAC). These findings are outstanding because no biomarker performs with 100% sensitivity and specificity in the history of cancer biomarker identification and validation, especially for an early stage of the disease. These findings remain to be validated before being applied in clinical settings because the GPC-1^+^ cross is also higher in others cancer such as breast cancer (Su et al., 2006). Studies about EVs have made great progress. However, there are still huge challenges concerning nerve diseases, especially in CNS diseases, due to the insufficiency of samples and contradictory conclusions. For instance, Winston et al. (2018) found that plasma exosomes containing neurogranin were highly diagnostic (sensitivity = 86.1 ± 0.037, confidence interval = 63.4–100) in distinguishing mild cognitive impairment patients from cognitively normal controls. However, a great number of published studies indicated that L1 cell adhesion molecule (L1CAM, CD147) positive exosome-containing Aβ42, T-tau, P-T181-tau, α-syn, and other proteins or ncRNA had higher diagnostic power for neurodegenerative diseases (Shi et al., 2014; Fiandaca et al., 2015; Yang et al., 2018; Jia et al., 2019; Jiang C. et al., 2020; Wang and Zhang, 2020). That said, the conclusions remain to be confirmed in longitudinal studies due to the small sample size. In addition, the enriched method of neuronal exosomes in immunoprecipitation with anti-L1CAM antibody further needs to be validated because the L1CAM protein is not only expressed in the nervous system but also expressed in other tissues or cells, such as soft tissue, fallopian tube, kidney cells, and epithelial cells (according to the Human Protein Atlas)^1^ (Pace et al., 2019; Asano et al., 2020), which may mean that the EVs pulled down with anti-L1CAM antibodies are not solely derived from neural tissues. Meanwhile, EVs derived from CSF are valuable for diagnosing CNS diseases (Jia et al., 2019; Muraoka et al., 2020). However, CSF samples are commonly obtained by lumbar puncture, which is an invasive test that can cause headaches and other side effects (Doherty and Forbes, 2014). Thus, it is challenging to recruit patients and healthy volunteers who agree to undergo lumbar puncture. Moreover, most research on the neuronal EVs as a biomarker has relied on the patient’s serum or plasma. Although serum or plasma is easily collected compared to CSF, the EVs in the serum or plasma is pool of EVs that come from various cells and organs. This factor may compromise the repeatability and accuracy of the results.

## Conclusion and future directions

During the past 5 years, EVs research has confirmed the transmission of EVs cargo between cells. Given the important role and therapeutic significance of EVs in neurological diseases, this topic is likely to continue under the spotlight.

One challenge for the field is that most studies were performed using a patient’s serum plasma or animal model. The limited availability of brain tissue currently constrains these studies. However, accumulating evidence demonstrates that isolating and purifying unmixed neuronal EVs from these samples is extremely difficult. Moreover, results from cultured primary neurons or cell lines as research objects *in vitro* are inconsistent due to the difference between protocols, operators, and devices. More importantly, *in vitro* studies cannot replicate exactly the state of physiology or pathology *in vivo*, which precludes a direct clinical translation. In the future, developing improved methods to isolate and purify EVs from neuropathological patients’ humor will be extremely beneficial for this field.

Another major challenge is understanding the underlying molecular mechanisms of packaging and transmission of neuronal EVs cargo. Analyzing the interaction of different trafficking proteins involved with both processes is extremely challenging. Although several studies found that many trafficking proteins and complexes contribute to the packaging and transmission of EVs cargo, both processes are unclear. Many proteins are associated with various cellular functions and participate in multiple pathways; the regulated mechanism remains to be investigated.

Despite the progress made, there are several key gaps in our knowledge. First, we do not yet know the physiological purpose of the generated EVs. Second, whether EVs maintain an essential cellular function is not known. Finally, although the different sizes of EVs have now been identified, how these different EVs are generated is unclear. The development of new technologies to track, isolate, and analyze EVs will be essential in addressing these important questions.

## Author contributions

ZJ and TZ designed the structure of the manuscript and finalized the manuscript. ZJ drafted the manuscript. ZH, NL, and YL provided critical revisions and improvements to the manuscript. All authors approved the final version of the manuscript.

## Footnotes

1
www.proteinatlas.org
